# A Case Report of Conservatively Managed Boerhaave Syndrome

**DOI:** 10.7759/cureus.55225

**Published:** 2024-02-29

**Authors:** Srushti S Mahant, Ajay Lanjewar

**Affiliations:** 1 Department of Medicine, Jawaharlal Nehru Medical College, Datta Meghe Institute of Higher Education & Research, Wardha, IND; 2 Department of Respiratory Medicine, Mahatma Gandhi Institute of Medical Sciences, Sevagram, IND

**Keywords:** esophageal perforation, mediastinitis, subcutaneous emphysema, vomiting, boerhaave syndrome

## Abstract

Boerhaave syndrome is an esophagal perforation due to a rupture of the esophagus wall caused by intense vomiting with mediastinitis and subcutaneous emphysema. It is a relatively rare and potentially life-threatening ailment that requires prompt diagnosis and treatment. This case presents an overview of the syndrome, including morbidity, mortality, and treatment strategy. In this case, a 56-year-old male presented to the hospital during emergency hours with shortness of breath, chest pain, and dullness in the neck and a history of binge alcohol abuse seven days ago, followed by a severe bout of vomiting. The patient was managed conservatively, requiring another hospitalization for surgery, and was later discharged from the hospital postoperatively without any complications.

## Introduction

Boerhaave syndrome is named after Herman Boerhaave, a Dutch physician who initially reported the illness in 1724 [1]. The syndrome usually develops after profound vomiting or retching, which causes a sudden and large increase in intraluminal pressure, resulting in esophageal rupture. Boerhaave syndrome has a high mortality, making early detection and treatment critical for avoiding complications [2]. Even if the patient is diagnosed early, the mortality is high. Conservative treatment should comprise intravenous fluids and broad-spectrum antibiotics that cover a wide range of both anaerobic and aerobic microorganisms, along with a proton pump inhibitor. To accelerate esophageal repair, nutritional assistance must be initiated either enterally or parenterally [3]. Without medical treatment, the prognosis for Boerhaave syndrome is only a few days. Because a lack of therapeutic interventions can be lethal, management emphasizes prompt recognition and intervention. Boerhaave syndrome is one of the rare lethal disorders where mortality reaches 100% if not treated early.

## Case presentation

**Figure 1:**
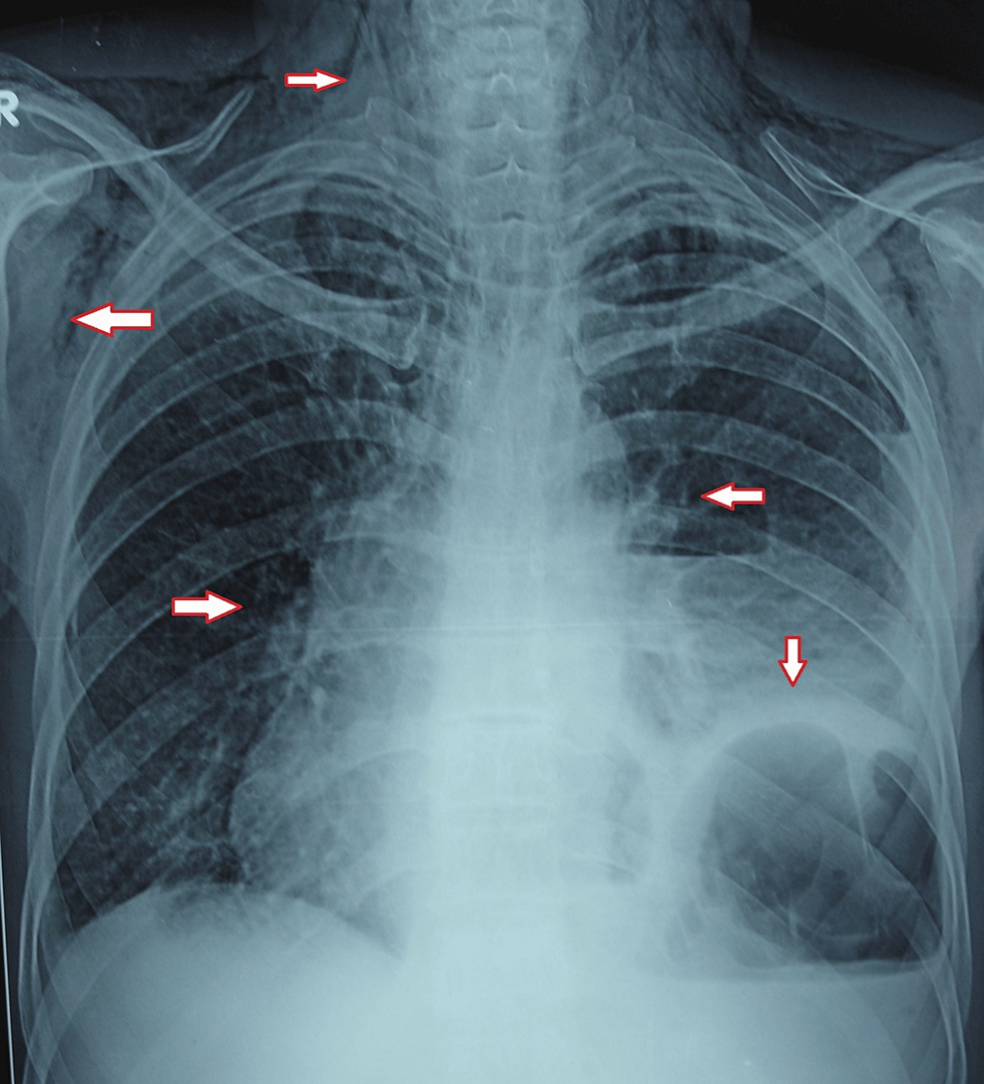
Chest X-ray showing a raised left dome of the diaphragm and air-fluid level with pleural collection along with subcutaneous emphysema in the neck, chest, and axilla.

**Figure 2:**
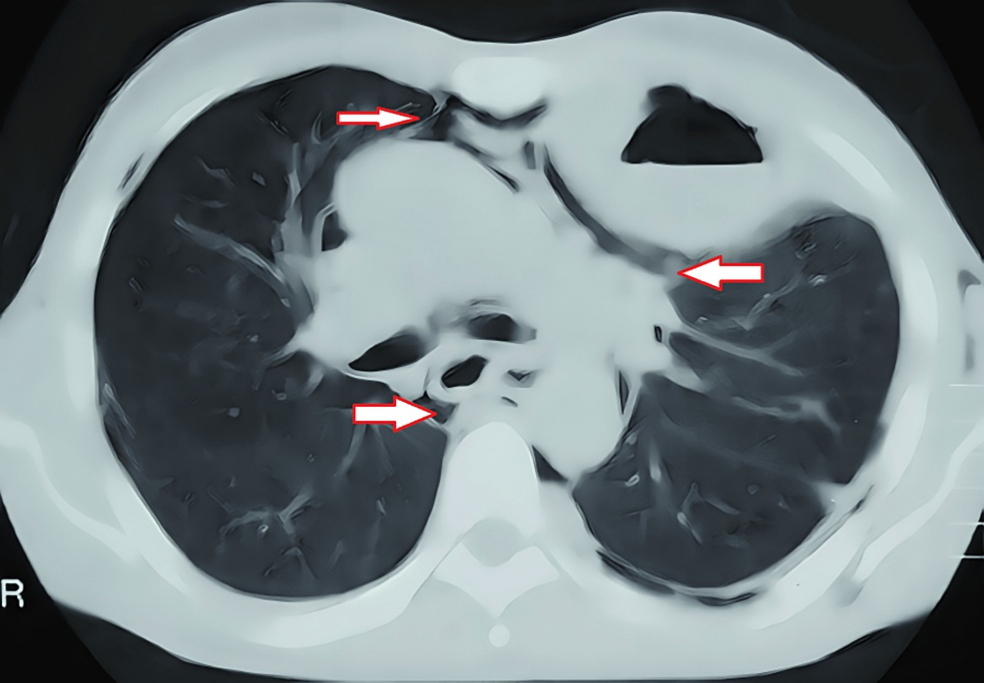
Computed tomography of the chest showing mediastinal emphysema with left loculated hydropneumothorax and subcutaneous emphysema around the trachea and neck.

A 56-year-old male was referred to the hospital during emergency hours as a case of pulmonary tuberculosis and lung abscess with subcutaneous emphysema with the presenting complaints of fullness in the neck, odynophagia, breathlessness at rest, abdominal and chest pain, inability to lie supine, orthopnea, and fever. The patient had a history of binge drinking seven days prior, which was followed by a severe bout of projectile vomiting twice and hoarseness of voice. There was no history of hematemesis, melena, or cough, with no comorbidities. He was on anti-Koch therapy as well as sorbitrate, antibiotics, and painkillers for seven days. On physical examination, the patient was restless with rigors. On room air, the SpO_2_ was 94%. Pallor, clubbing, cyanosis, or edema were absent. The clinical examination was suggestive of decreased chest movements on the left side with crepitations all over the chest, more on the left side; moreover, the neck area was involved up to the mandible. On auscultation, bilateral rhonchi were heard with reduced breath sounds on the left side in the inframammary area, intra-axillary area, and infrascapular area. A chest X-ray revealed subcutaneous emphysema, pneumomediastinum, and loculated pleural effusion over the left side of the chest (Figure 1). A computed tomography (CT) scan of the chest revealed a left loculated hydropneumothorax as well as subcutaneous emphysema along with pneumomediastinum (Figure 2).

**Figure 3:**
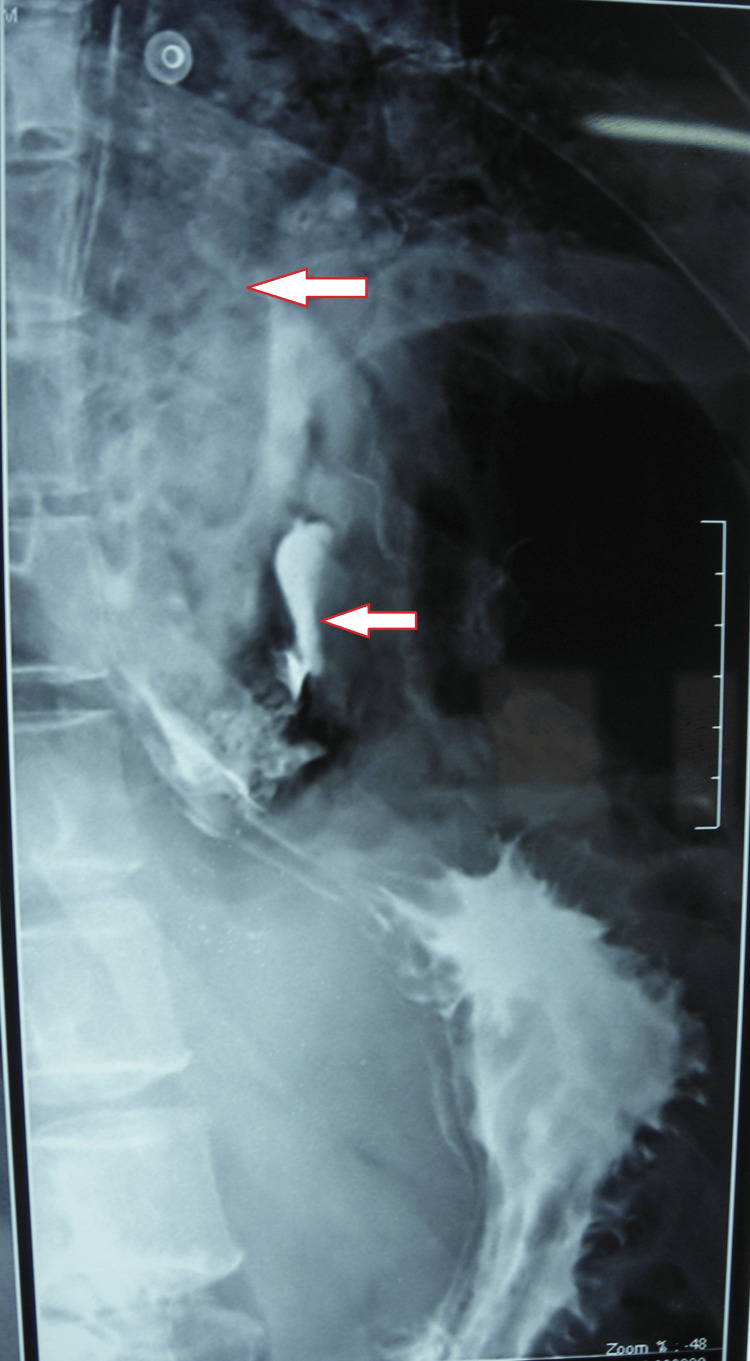
Gastrografin study with non-ionic oral contrast showing the leak in the mediastinum on the left side 1-2 cm above the gastroesophageal junction.

The next day, a gastrografin study was done with non-ionic oral contrast, which confirmed the leak in the lower part of the esophagus on the left side 1-2 cm above the gastroesophageal junction (Figure 3). Subsequently, a surgical opinion was sought, and as per their advice, the patient was kept nil by mouth, and a percutaneous endoscopic gastrostomy (PEG) feeding tube was inserted. He was suffering from mediastinitis and septicemia, and already it was the eighth day, so surgical repair was not advisable.

**Figure 4:**
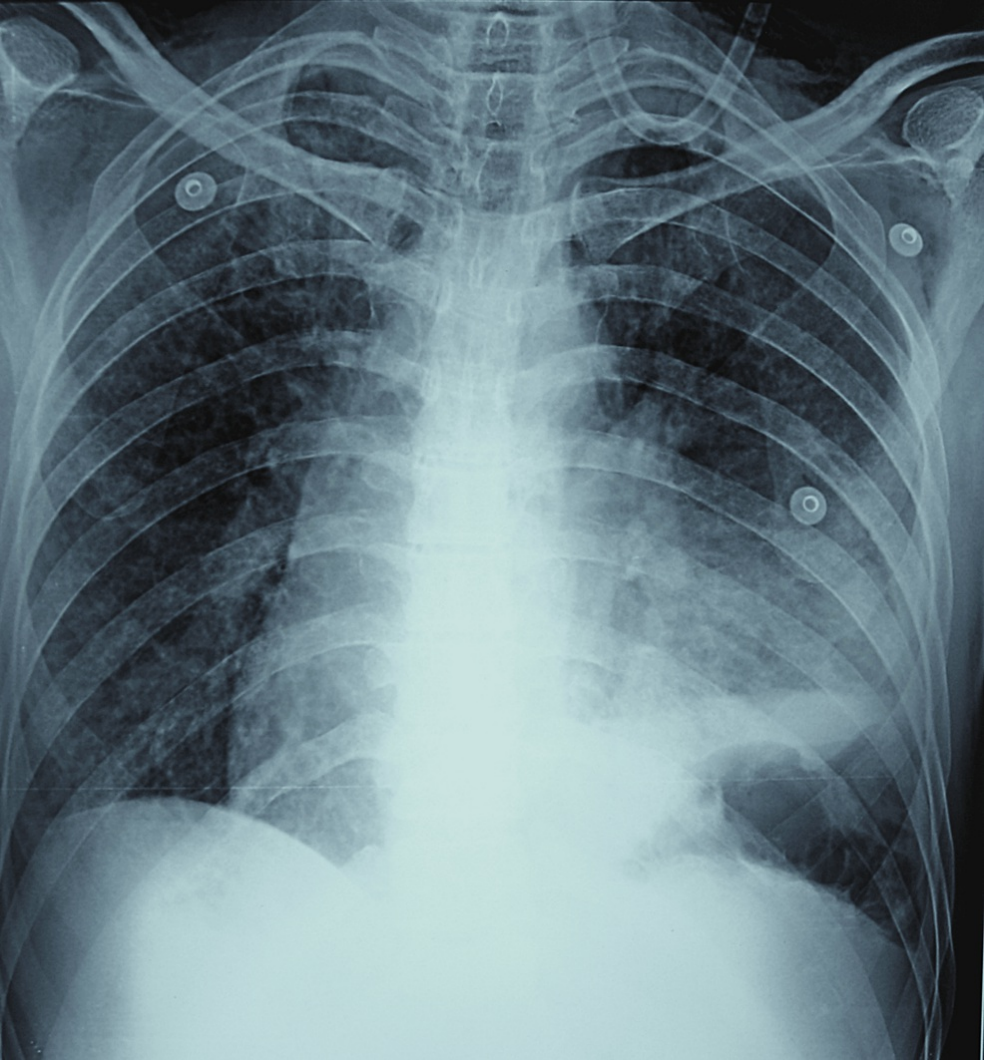
Post-tapping (thoracocentesis) chest X-ray showing reduced emphysema.

**Figure 5:**
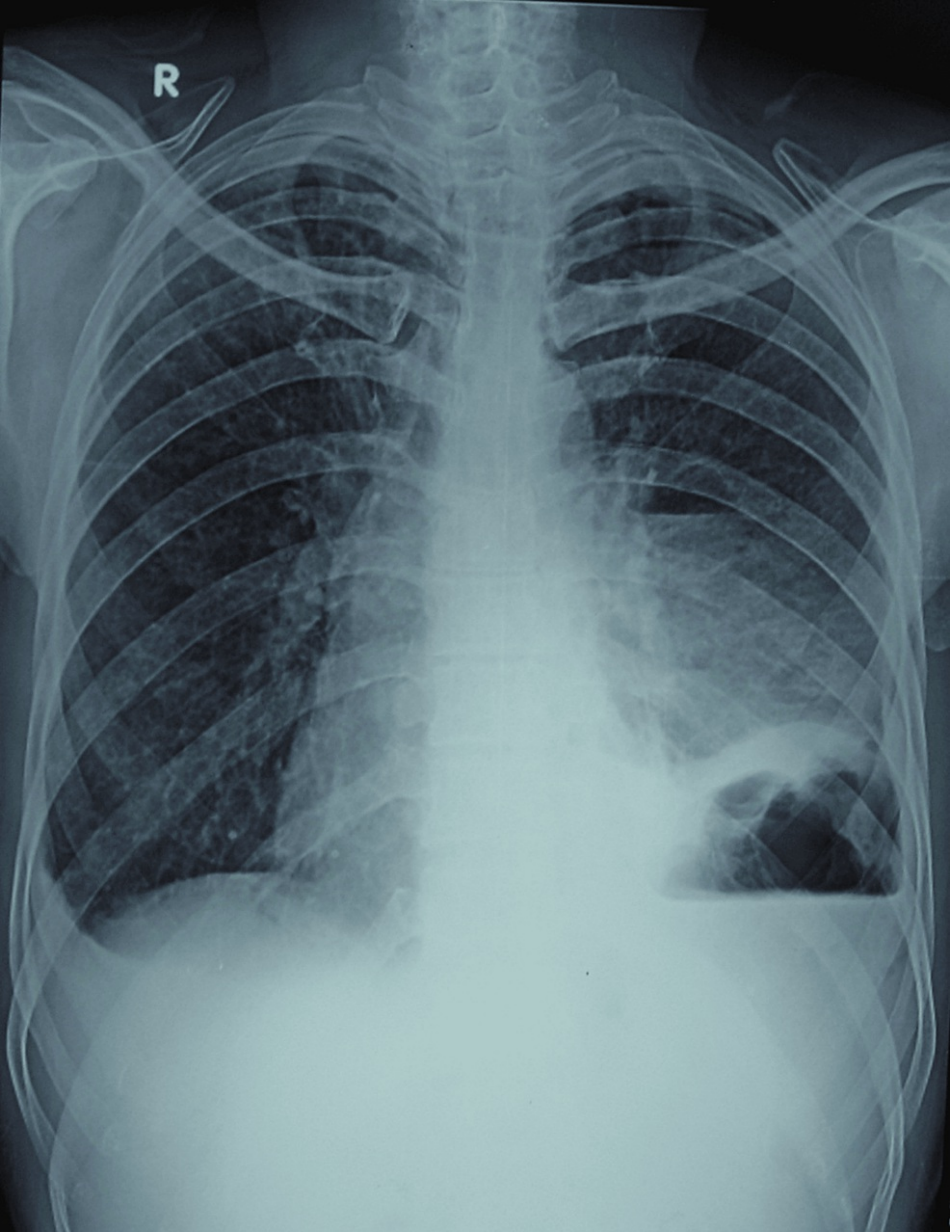
Chest X-ray showing reduced subcutaneous emphysema and pleural collection.

The lab investigations were as follows: hemoglobin of 13.3 g/dL, normal complete blood count, negative retrovirus, and normal liver function tests, kidney function tests, and electrolytes. Meanwhile, pleural aspiration was done, which showed exudative pleural effusion. The pleural fluid analysis report showed 120-130 mL yellow, turbid fluid exudate with a total protein of 4.6 g/dL, sugar of 29 mg/dL, amylase of 30 U/L, total cell count of 2,400 cells/cm, polymorphs of 89%, and lymphocytes of 11%. Ziehl-Neelsen stain and Gram stain were negative. Post-tapping (thoracocentesis), the emphysema was also reduced (Figure 4). Injections and broad-spectrum antibiotics, cefaparazin and sulbactam, along with amikacin and clindamycin with gram-negative and anaerobic coverage, were administered. On the 10th day of hospitalization, a follow-up gastrografin study was done that did not reveal the perforation, i.e., it had been healed; moreover, the subcutaneous emphysema and pleural collection were reduced, as seen on the chest X-ray (Figure 5). The treatment was continued with oxygen support, and after a few days, the PEG feeding tube was removed.

**Figure 6:**
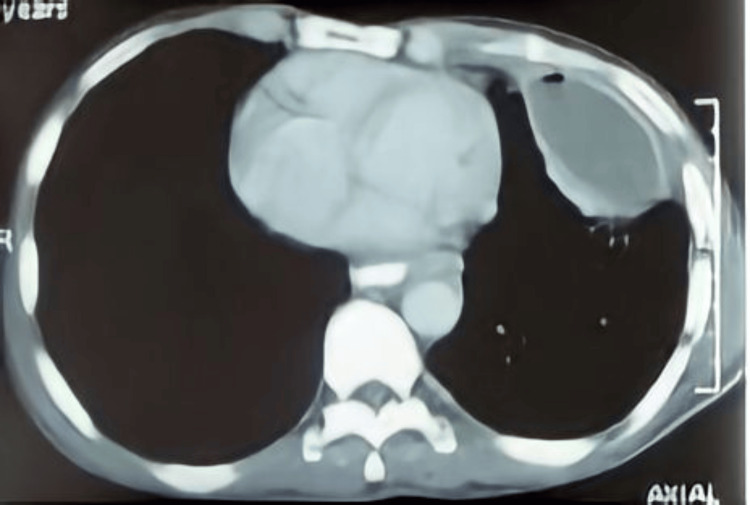
Computed tomography of the chest showing loculated empyema.

**Figure 7:**
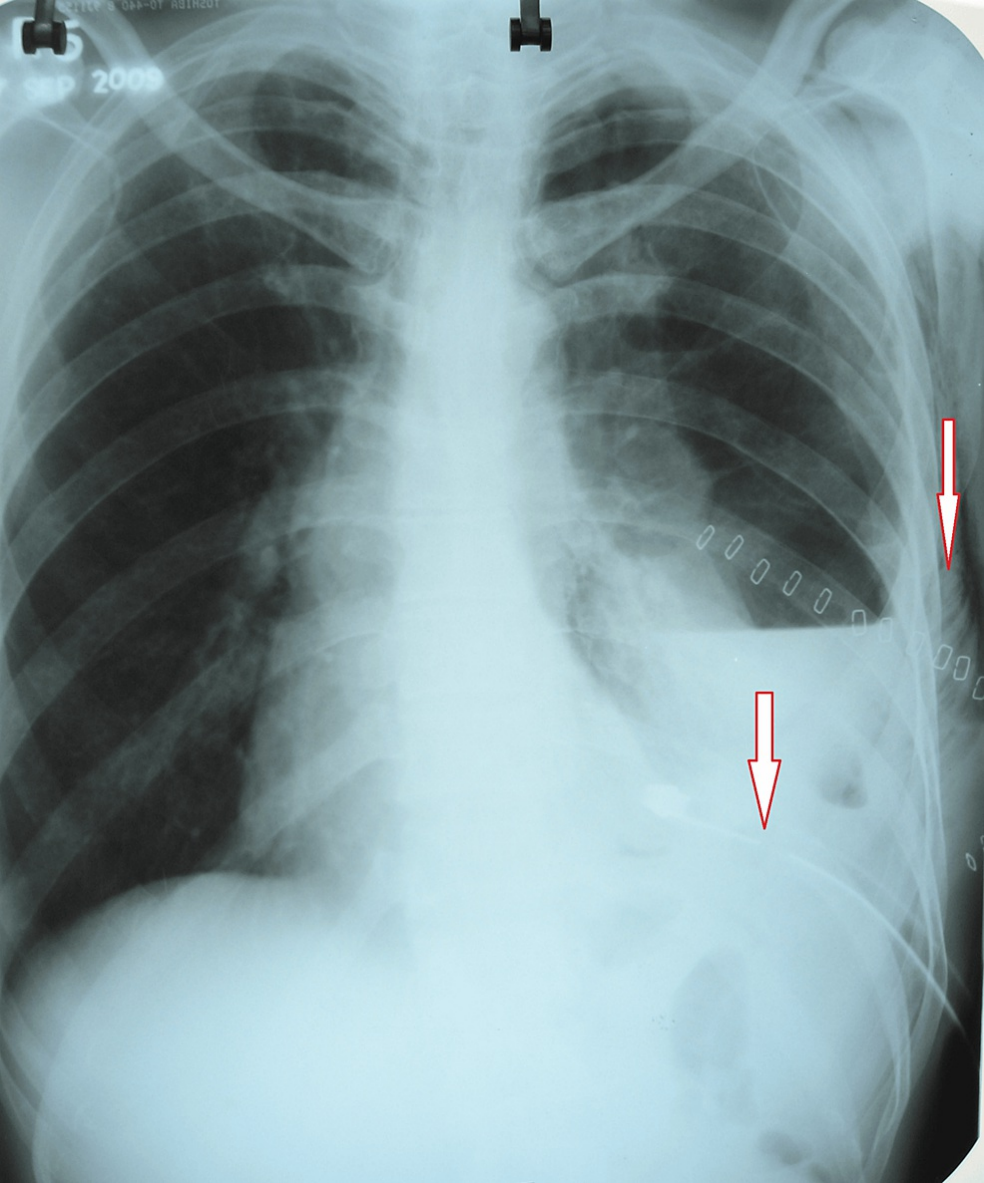
Postoperative chest X-ray showing intercostal drainage in situ for loculated pyopneumothorax with visible pleural thickening.

He was discharged on oral antibiotics. After five weeks, he presented with symptoms of cough, persistent chest pain, and fever. A repeat CT scan revealed loculated empyema (Figure 6). He underwent video-assisted thoracoscopic surgery, and decortication was done. Figure 7 shows the postoperative chest X-ray with intercostal drainage in situ for loculated pyopneumothorax with visible pleural thickening.

**Figure 8:**
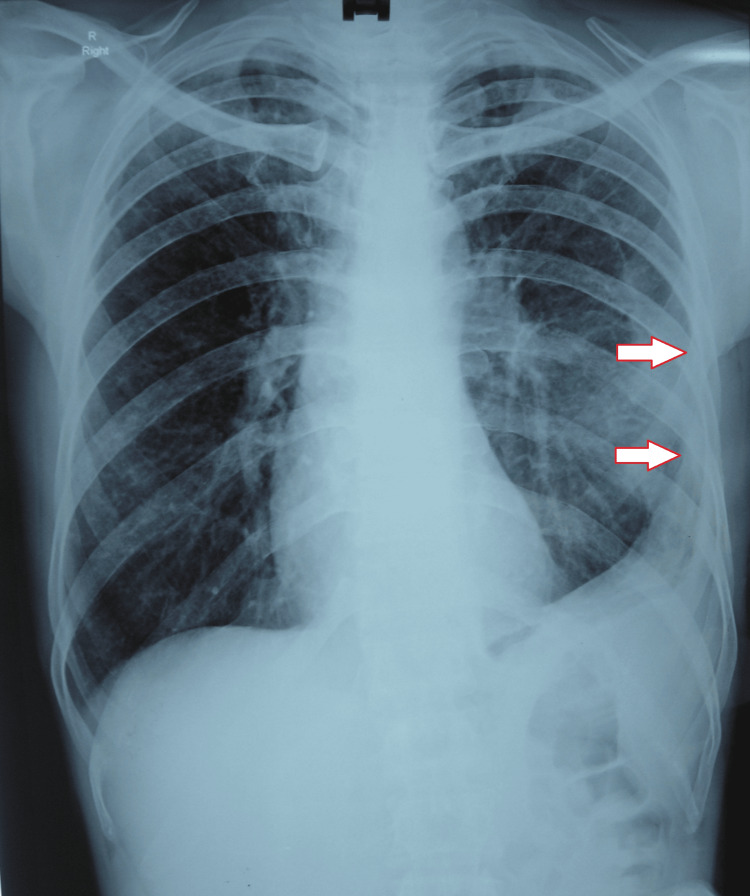
Postoperative chest X-ray showing left pleural thickening with no pleural collection.

**Figure 9:**
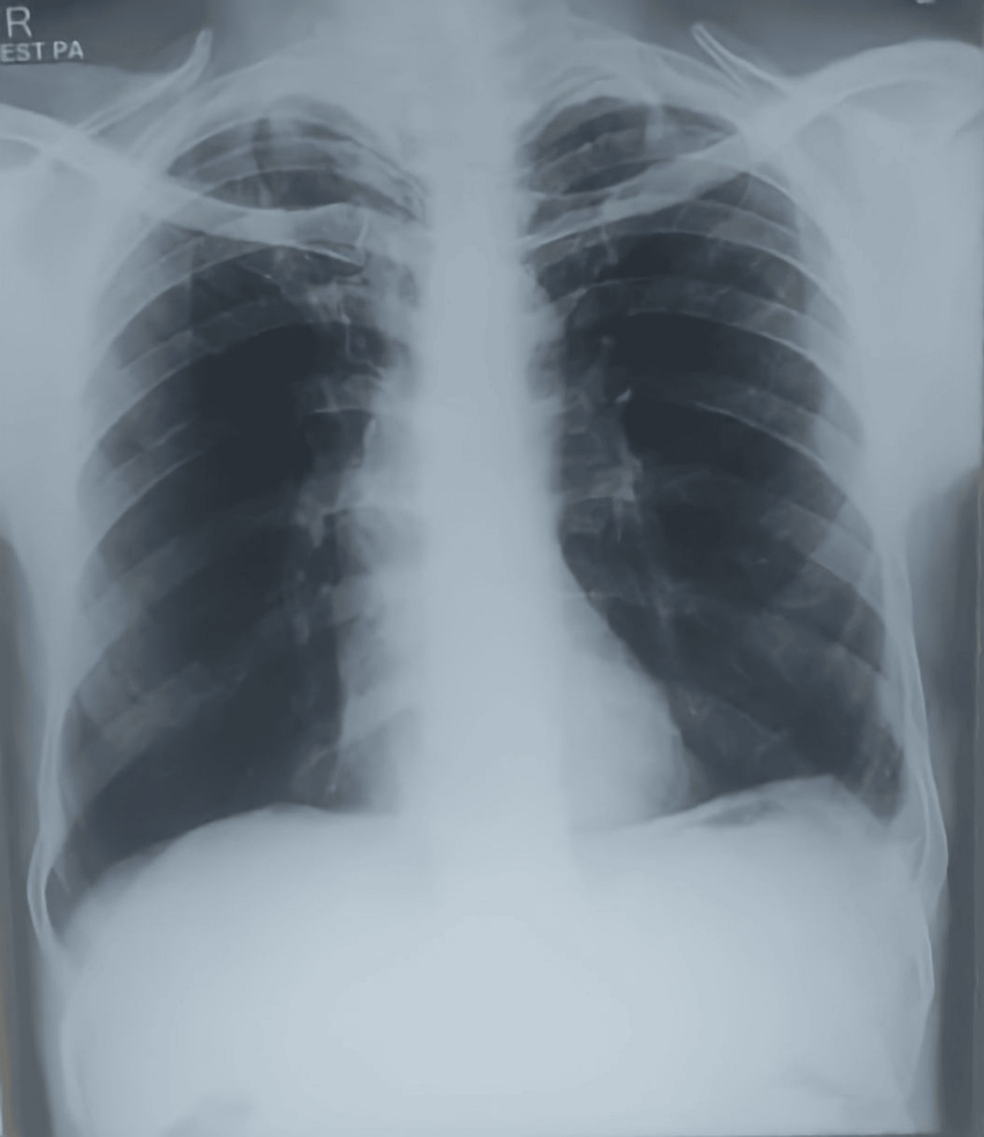
Follow-up chest X-ray after six months showing good clearance of empyema.

He responded very well, and his postoperative collection was nil with left pleural thickening, as seen on chest X-ray (Figure 8). Coincidentally, he was followed up after six months, with a chest X-ray showing no evidence of empyema (Figure 9).

**Figure 10:**
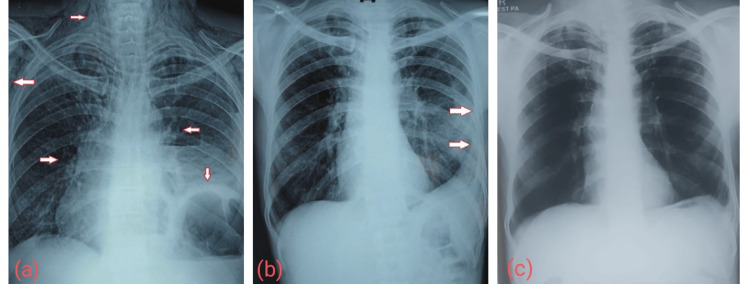
(a) Chest X-ray at presentation showing a raised left dome of the diaphragm and air-fluid level with pleural collection along with subcutaneous emphysema in the neck, chest, and axilla. (b) Postoperative chest X-ray showing left pleural thickening with no pleural collection. (c) Follow-up chest X-ray after six months showing good clearance of empyema.

A comparison of chest X-rays at presentation and post-intervention to assess the prognosis and improvements is presented in Figure 10.

## Discussion

Boerhaave syndrome is a relatively rare and potentially life-threatening illness that requires immediate diagnosis and early surgical intervention. The diagnosis is difficult because there are no classic symptoms and delays in presentation for medical care. Spontaneous lesions can affect only a part of the esophageal wall, i.e., mucosal, leading to Mallory-Weiss syndrome, or may cause full-thickness rupture, i.e., transmural, with communication from the lumen of the esophagus to the pleural cavity, leading to Boerhaave syndrome [4]. The physiology behind esophageal rupture is a sudden increase in intraluminal pressure caused by vomiting, resulting from neuromuscular incoordination causing the cricopharyngeus muscle sphincter within the esophagus to relax [5]. Excessive eating and/or alcohol intake are frequently related to the illness. The tear in Boerhaave syndrome is most commonly found in the left posterolateral wall of the lower portion of the esophagus, approximately 2-4 cm above the stomach [2]. The classic triad (Mackler’s triad) of vomiting, chest discomfort, and subcutaneous emphysema is present in a mere 50% of cases, and early diagnosis requires a high index of suspicion [1,3]. Forceful vomiting commonly causes a Mallory-Weiss mucosal tear, but it is unusual for the entire wall of the esophagus to rupture (Boerhaave syndrome) [6].

The preferred diagnostic modality is CT of the chest, and expeditious surgical intervention is the fundamental cornerstone of treatment. Plain chest radiography supports the diagnosis of Boerhaave syndrome, which is confirmed by a CT scan of the chest. In patients suffering from Boerhaave syndrome, a plain chest radiograph is almost always abnormal, with mediastinal or free pleural air as the initial radiologic manifestation. Pleural effusion(s) with or without pneumothorax, widened mediastinum suggestive of air, and mediastinitis, along with subcutaneous emphysema, commonly develop hours to days later. CT scans may reveal air in the extra-esophageal region and fluid in the peri-esophageal region, including or excluding gas bubbles. Moreover, mediastinal enlargement, along with air and fluid in the pleural spaces, can be detected on CT scans [7]. The mortality rate for Boerhaave syndrome ranges from 20% to 40%, and the presence of comorbidities such as alcohol abuse and cirrhosis increases the risk of morbidity and mortality [8-10]. The higher prevalence of differential diagnoses, such as perforated gastric or duodenal ulcer, acute myocardial infarction, pericarditis, pneumothorax, pulmonary thromboembolism, diaphragmatic hernia, dissecting aortic aneurysm, and acute pancreatitis, frequently impedes early diagnosis. Persistent vomiting may raise suspicions of acute pancreatitis, while left-sided chest pain radiating to the left shoulder may raise suspicions of aortic dissection or myocardial infarction, and subcutaneous emphysema may raise suspicions of traumatic pneumothorax in patients with barotrauma on mechanical ventilation, all of which may mislead clinicians about the underlying Boerhaave syndrome [5,10]. The potential delay in confirming a diagnosis elucidates the excessive mortality associated with the pathology, ranging from 10% to 25% for cases treated within the initial 24 hours and escalating to 40%-60% for cases treated beyond the 48-hour timeframe. Management relies on rapid recognition [11,12]. If left untreated, mortality is 100%.

Boerhaave syndrome necessitates an endoscopic approach for cases diagnosed within 48 hours, provided there are no indications of sepsis. However, if a patient is diagnosed within the initial 48 hours and presents with sepsis, surgical intervention in the form of thoracotomy with hemifundoplication and pleural/mediastinal drainage is recommended. In instances where diagnosis occurs after the initial 48 hours, a conservative treatment approach is advised. Surgical intervention is only warranted in the presence of a septic profile in such cases [13]. As Boerhaave syndrome is a rare presentation and has a high degree of suspicion, for any patient presenting with symptoms of forceful vomiting, chest discomfort, and a radiological investigation showing mediastinitis and sudden-onset subcutaneous emphysema over the neck, extending into the chest wall, arms, and even face, immediate treatment must be provided along with conservative management to improve survival.

## Conclusions

Boerhaave syndrome, being a relatively rare but potentially fatal illness, requires prompt diagnosis and early surgical intervention. Early diagnosis and treatment are essential for preventing complications and enhancing outcomes. A significant degree of suspicion is required, particularly for patients with a history of alcoholism. If the patient survives for a few days, then conservative management, including very close observation of symptoms and prompt detection of signs, radiological investigation, and treatment modality, either medical or surgical, by multidisciplinary discussion, is the key to excellent outcomes. As patients with projectile vomiting and subcutaneous emphysema secondary to mediastinitis or pneumomediastinum are unrecognized, and ingestion of food if continued, and delay in prompt surgical repair and conservative management leads to unpublished deaths, our main purpose in publishing this case of maintaining a high degree of suspicion and vigorous management is lifesaving.
